# Urban-Rural Differences in Cardiovascular Disease Risk Factors: A Cross-Sectional Study of Schoolchildren in Wuhan, China

**DOI:** 10.1371/journal.pone.0137615

**Published:** 2015-09-09

**Authors:** Kayne McCarthy, Long-biao Cai, Fang-rong Xu, Pei-gang Wang, Hong-liang Xue, Yong-ling Ye, Shang-zhi Xiong, Zhao-min Liu, Qi-qiang He

**Affiliations:** 1 School of Public Health, Wuhan University, Wuhan, P. R. China; 2 University of Hawaii Office of Public Health Studies, Honolulu, Hawaii, United States of America; 3 School of Public Health and Primary Care, The Chinese University of Hong Kong, Hong Kong, China; 4 Global Health Institute, Wuhan University, Wuhan, P. R. China; McMaster University, CANADA

## Abstract

**Background:**

China’s rapid population growth and urban migration has developed healthcare inequity across the urban-rural divide. Past studies comparing cardiovascular disease (CVD) risk factor prevalence amongst urban-rural Chinese children are sparse and conflicting. We examined the association between urban-rural residence and risk of offspring CVD in Chinese children.

**Methods:**

A cross-sectional study was conducted in Wuhan, China, during May and June 2010. CVD risk factors include; waist circumference (WC), systolic blood pressure (SBP), diastolic blood pressure (DBP), fasting blood glucose (FBG), triglycerides (TG), high-density lipoprotein (HDL) cholesterol, low-density lipoprotein (LDL) cholesterol, body mass index (BMI), cardiorespiratory fitness (CRF), metabolic syndrome (MetS), and metabolic risk score (MRS). Analysis of covariance and multivariable logistic regression were used to estimate associations between urban-rural residence and offspring CVD risks.

**Findings:**

A total of 579 Chinese children (338 boys and 241 girls) aged 9.6 (0.7) years participated in this study. Rural boys had significantly lower CRF and higher FBG, TG, and MRS, while urban boys had significantly higher LDL and DBP. Rural girls had significantly higher BMI, FBG, and TG, as well as lower CRF. Rural children were at increased risks for decreased CRF, elevated MRS, and TG, (OR:2.04, 95%CI:1.29–3.25), (OR:2.33, 95%CI:1.50–3.62), and (OR:2.40, 95%CI:1.62–3.57), respectively. Rural girls and mothers were at increased risks for overweight(OR:7.19, 95%CI:1.64–31.6)/obesity (OR:1.683, 95%CI:1.01–2.82). However, rural boys and fathers were less likely to have overweight(OR:0.62, 95%CI:0.34–1.12)/obesity (OR:0.68, 95%CI:0.48–0.97).

**Conclusions:**

Rural residence was significantly associated with increased CVD risks amongst Chinese children. It is important to provide interventions aiming at China’s urban-rural healthcare inequity and community-based approaches that reduce familial CVD risk.

## Introduction

Unprecedented as China’s population growth over the past two decades, the rural to urban migration within China is likely the largest in history with over 145 million rural-to-urban immigrants [1]. Population growth coupled with migration to urban centers has developed vast healthcare inequity across China’s urban-rural divide. In 2009, the healthcare expenditure per capita between urban and rural areas was 2176.6¥ (351.8 USD) and 562.0¥ (90.8 USD) [2], respectively. Furthermore, the ratio between urban and rural healthcare technicians is another representation of substantial healthcare inequity, with 7.62 healthcare technicians per 1000 people in urban areas and 3.04 per 1000 people in rural areas [2].

Cardiovascular health across the urban-rural divide is not clearly understood. Several studies have found that cardiovascular disease (CVD) risk factors generally increase in prevalence amongst rural residents [3–5], while other studies have conflicting findings [6, 7]. In China, urban-rural cardiovascular health research has primarily focused on adults, while studies assessing cardiovascular health amongst children are limited. Only one study, to our knowledge, has compared CVD risk factors amongst children between rural and urban districts [8]. Utilizing the International Diabetes Federation’s (IDF) classification of children metabolic syndrome (MetS) [9], this study found that rural children, aged 6 to 9 years old, had significantly higher prevalence of elevated blood pressure (BP), triglycerides (TG), and fasting blood glucose (FBG), while urban children exhibited elevated cholesterol and weight status.

In order to provide further depth of understanding related CVD risk factors amongst Chinese children, the present cross-sectional study was conducted. We assessed the association between urban-rural residence and an expansive set of CVD risk factors and the aggregation of CVD risk factors using MetS [10] and the continuous metabolic risk score (MRS) [11]. Further analyses were performed to examine the association between urban-rural residence and parental weight status, to expound on potential urban-rural intergenerational CVD risks.

## Methods

### Study subjects

We conducted a cross-sectional study in Wuhan, China, from May to June 2010. The study design and methods have been published previously [12–14]. We recruited a representative sample by a multi-stage sampling method. Two districts were randomly selected within the urban and rural districts in Wuhan city, respectively. And then one primary school was randomly selected in each district. The original design of this study was a 3-year prospective cohort study. Therefore, we recruited students in the 3^rd^ and 4^th^ grades so that they can be followed up for additional 2 years. Written informed consent was obtained from parents of the children. The Medical Research Ethics Committee of Wuhan University and the University of Hawaii Human Subjects Institutional Review Board approved the study.

### Measures

The children’s standing height, weight, and waist circumference (WC) were measured by trained investigators. Body mass index (BMI) was calculated by dividing weight (kg) by height squared (m^2^). A trained technician measured BP with all children sitting in an upright position for at least 5 min. The mean of two measurements taken in the morning was used for data analysis. The mean arterial pressure (MAP) was calculated as diastolic blood pressure (DBP) + [(systolic blood pressure (SBP)–DBP)/3]. Pubertal development was assessed by direct observation according to the Tanner stages. Breast development in girls and genital development in boys were used for pubertal classification [15]. Parents of the children reported their age, gender, height, weight and education using a questionnaire.

### Blood Samples

After an overnight fast, blood samples were drawn from the antecubital vein. FBG, high-density lipoprotein (HDL), low-density lipoprotein (LDL), and fasting TG, were analyzed enzymatically at the Wuhan Center for Disease Control and Prevention with a Mairui BS-300 Automatic Analyzer (Mairui High Technologies Corp. Shenzhen, China).

### Metabolic Syndrome

Metabolic syndrome (MetS), defined as the aggregation of cardiovascular risk factors [16], is a robust and commonly used aggregate variable in the assessment of CVD risk in children and adult populations [10, 12, 17]. MetS was defined as ≥3 of the following criteria proposed by De Ferranti et al [10]: WC ≥75th percentile (gender specific for Chinese children) [18], TG ≥1.1 mmol/L (100 mg/dL), HDL <1.2 mmol/L (45 mg/dL) in boys and <1.3 mol/L (50 mg/dL) in girls, FG ≥6.1 mmol/L (110 mg/dL), and SBP and DBP ≥90th percentile (age- and gender-specific cutoff points for Chinese children) [19].

### Metabolic Risk Score

CVD risk factors (WC, MAP, HDL, TG, and FG) were used to compute the Metabolic Risk Score (MRS). First, each risk factor was standardized as follows: standardized value = (value—mean)/ standardized deviation. The HDL scores were multiplied by -1 because it is inversely related to metabolic risk. Next, the MRS was calculated as the sum of the five scores. The higher MRS score indicates a poorer metabolic profile [11].

### Cardiorespiratory Fitness

Cardiorespiratory fitness (CRF) was determined by the 20-meter shuttle-run test [20]. This validated test is a useful measure of aerobic capacity in children [21]. Children were asked to run back and forth on a 20-meter course at a pre-determined speed guided by audio signals from a CD player. The frequency of sound signals was increased at a rate corresponding to an increase in running speed of 0.5 km/h each minute, from a start speed of 8.5 km/h. The running speed increased progressively after each minute, and the subjects entered a new level. Groups of six children were instructed to run at speeds following the audio signal and to complete as many as laps as possible, until they could not cope. The children were stopped when they could not follow the signal any more. Predicted maximum oxygen uptake (VO_2max_) derived from the level (maximal speed) and number of laps in the test was used as a measure of CRF.

### Quality Control

All field workers participated in a training program prior to the study. The anthropometric measurements were performed using standard methods. Blood pressure was measured using mercury sphygmomanometer according to the standard procedures by trained technicians. Laboratory analyses were also subject to strict quality control. All of the questionnaires were double-checked by staffs. Up to three attempts were made to contact parents by telephone to fill in missing information.

### Statistical Analysis

Parents’ BMI was calculated using their self-reported height and weight. They were then categorized into normal or overweight (OW)/obese (OB) groups based on Chinese cut-off points [22]. The Chinese age- and gender- specific BMI and WC [23] cut-off points were used to define general obesity and central obesity among children. The characteristic differences between urban and rural groups were assessed using ANOVA and Chi-square test, where appropriate. Analysis of covariance was conducted to compare CVD risk factors between different residences. Multivariate logistic regression was used to estimate the associations between residence and each of the CVD risk factors, after adjusting for several confounding variables including age, gender, pubertal stage, paternal education, parental smoking and parental BMI [13, 24, 25]. Paternal education has been commonly used as a surrogate for income [26]; therefore, it was employed as a proxy for socioeconomic status (SES) in the present study. Further analysis, using multivariate logistic regression, estimated the association between rural residence and offspring weight status, parental weight statuses, MRS, and CRF. For CRF and MRS, both continuous variables and tertile median splits were used to measure association with urban-rural residence. For both variable tertiles groups, higher tertiles indicate elevated CVD risk (low CRF and high MRS). Data analyses were conducted using the SPSS statistical package (version 20.0; SPSS Inc, Chicago, Ill. USA).

## Results

**Fig 1:**
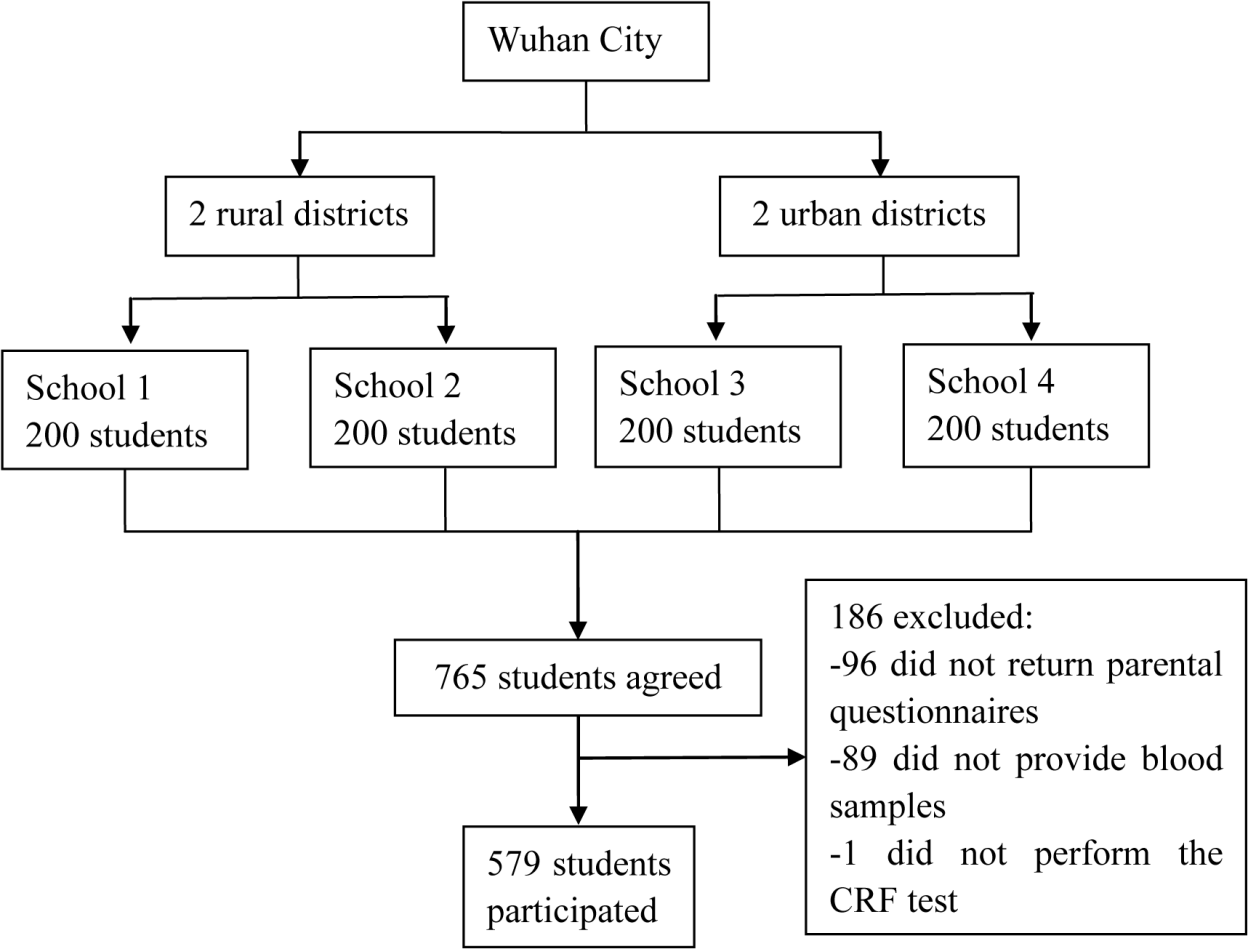
Flow of the participants through the study.

Of 800 children who were invited to participate in the study, 765 (95.6%) agreed to participate. After excluding 186 children with missing data (96 did not return parental questionnaires, 89 did not provide blood samples and 1 did not perform the CRF test), 579 (72.4%) were included in the final analysis (Fig 1). Assuming α  =  0.05 and the power of the study (1-β) =  0.80, 560 participants would be needed to detect a difference of 2 standard deviation in VO_2_max between normal weight and obese children. Given that respondents might refuse to participate or drop out from this study, we chose to recruit 800 students. As 579 children were included, the sample size is adequate for the present study. Compared with children who were excluded from this study, the participants were slightly younger and had non-significantly lower BMI.

The characteristics of the study populations are shown in Table 1. No significant differences were found in age, gender, mother age, and parental smoking between the two residence groups. Increased puberty stage, father age, and parental education status, were more prevalent in the urban group.

**Table 1:** Characteristics of the participants^a^.

	Urban Residence (n = 294)	Rural Residence (n = 285)	*P*-Value
Age, yr, mean (SD)	9.59 (.68)	9.60 (.67)	0.838
Gender, n (%)			0.274
Boy	165 (56.1)	173 (60.7)	
Girl	129 (43.9)	112 (39.3)	
Puberty Stage, n (%)			
Stage 1	0 (0.0)	8 (2.8)	<0.001
Stage 2	201 (68.4)	248 (87.0)	
Stage 3	92 (31.3)	29 (10.2)	
Stage 4	1 (0.3)	0 (0.0)	
Father Age, yr, mean (SD)	38.7(5.5)	37.1(3.14)	<0.001
Mother Age, yr, mean (SD)	35.6(3.4)	35.1(3.6)	0.105
Father Education, n (%)			0.002
Primary or below	6 (2.0)	14 (4.9)	
Middle School	68 (23.1)	95 (33.3)	
High school	128 (43.4)	97 (34.0)	
College or above	85 (28.9)	61 (21.4)	
Mother Education, n (%)			.009
Primary or below	18 (6.1)	12 (4.2)	
Middle School	84 (28.6)	113 (39.6)	
High school	115 (39.1)	83 (29.1)	
College or above	63 (21.4)	48 (16.8)	
Father smoking, n (%)	179 (60.9)	168 (58.9)	0.672
Mother smoking, n (%)	5 (1.7)	2 (0.7)	0.451

SD: standard deviation.

^a^There were some missing values.

Table 2 displays the adjusted means of CVD risk factors amongst the rural and urban children. Urban boys had significantly higher LDL and DBP, in comparison to their rural counterparts. Rural boys had significantly higher FBG, TG, MRS, as well as lower CRF. Rural girls had significantly higher BMI, FBG, TG, and MRS, as well as lower CRF, in comparison to urban girls.

**Table 2:** Participants’ CVD risk factors among different residence ^a^.

	Urban Residence (Mean ± SE)	Rural Residence (Mean ± SE)	*P*-Value
BMI (kg/m^2^)			
Boys	17.14 ± .21	17.10 ± .21	.881
Girls	15.76 ± .24	17.14 ±.27	.001
Total	16.63 ± .16	17.15 ± .16	.180
WC (cm)			
Boys	59.82 ± .65	60.46 ±.65	.490
Girls	57.09 ± .66	58.76 ±.74	.133
Total	59.07 ± .79	60.01 ± .79	.400
FBG (mmol/l)			
Boys	4.32 ± .03	4.54 ± .03	< .001
Girls	4.29 ± .04	4.50 ± .04	.001
Total	4.31 ± .04	4.51 ± .04	<0.001
HDL (mmol/l)			
Boys	1.23 ± .01	1.22 ± .01	.356
Girls	1.23 ± .01	1.22 ± .01	.640
Total	1.23 ± .01	1.23 ± .01	.892
LDL (mmol/l)			
Boys	2.44 ± .02	2.34 ± .02	< .001
Girls	2.44 ± .03	2.39 ± .03	.346
Total	2.42 ± .03	2.40 ± .03	.627
TG (mmol/l)			
Boys	0.91 ± .04	1.12 ± .04	.001
Girls	0.95 ± .05	1.13 ± .06	.040
Total	0.93 ± .06	1.05 ± .06	.140
SBP (mmHg)			
Boys	93.00 ± .77	92.39 ± .77	.575
Girls	89.01 ± .98	89.20 ± 1.10	.906
Total	91.4 ± 1.06	92.26 ± 1.05	.566
DBP (mmHg)			
Boys	60.43 ± .51	57.64 ± .51	< .001
Girls	57.52 ± .82	56.75 ± .91	.567
Total	58.74 ± .79	57.85 ± .78	.423
CRF (ml/kg/min)			
Boys	47.00 ± .28	45.97 ± .28	.008
Girls	45.96 ± .29	43.92 ± .33	< .001
Total	46.16 ± .34	45.08 ± .34	.026
MRS			
Boys	-.13 ± .20	.75 ± .20	.003
Girls	-.95 ± .26	.20 ± .29	.009
Total	-.40 ± .28	.44 ± .27	.030

Abbreviation: BMI, body mass index; CRF, cardiorespiratory fitness; CVD, cardiovascular disease; DBP, diastolic blood pressure; FBG, fasting blood glucose; HDL, high-density lipoprotein; LDL, low-density lipoprotein; MRS, metabolic risk score; SBP, systolic blood pressure; TG, triglycerides; WC, waist circumference.

^a^ Adjusted for gender (in total), age, puberty stage, and SES.

The associations between location and CVD components are presented in Table 3. Rural girls and rural boys were at significantly elevated odds of having hypertriglyceridemia, (OR:2.20, 95%CI:1.01–4.74) and (OR:2.94, 95%CI:1.75–4.97) respectively. The total rural group had a marginally significant increase in odds for having hypertensive SDP (OR:1.92, 95%CI:0.99–3.73). MetS was not significantly associated with urban or urban groups. The direction of association between urban-rural residence and weight status differed by gender amongst both offspring and parents. Compared with those in the urban communities, rural girls had a significant increased risk for OW/OB classification (OR: 5.64, 95%CI:1.28–24.95), while odds for elevated weight status amongst mothers were marginally significant (OR: 1.68, 95%CI:0.99–2.80). Unlike rural children and mothers, rural fathers had decreased odds for being overweight/obese (OR: 0.68, 95%CI:0.48–0.97). Although statistically insignificant, rural sons had similar point estimates to rural fathers (OR:0.62, 95%CI:0.34–1.12).

**Table 3:** Associations between location and CVD components.

CVD risk Factor	OR	95%CI
Central Obesity (WC)		
Girls	1.23	.26–5.76
Boys	.93	.48–1.80
Total	.98	.55–1.75
DBP		
Girls	1.33	.32–5.50
Boys	1.15	.27–4.93
Total	1.02	.41–2.58
SBP		
Girls	.94	.12–7.24
Boys	2.06	.96–4.40
Total	1.92	.99–3.73
Hypertriglyceridemia (TG)		
Girls	2.19	1.01–4.74*
Boys	2.95	1.75–4.97**
Total	2.51	1.68–3.75**
High LDL		
Girls	.67	.23–2.0
Boys	.901	.43–1.88
Total	.877	.49–1.56
High HDL		
Girls	1.02	.46–2.29
Boys	.92	.55–1.55
Total	.95	.62–1.45
MetS		
Girls	1.45	.32–6.54
Boys	1.40	.63–3.14
Total	1.45	.75–2.82
Offspring general obesity (BMI)		
Girls	5.64	1.28–24.95*
Boys	.616	.34–1.12
Total	.702	.43–1.15
Parental OW/OB		
Mother	1.68	.999–2.80
Father	.68	.48-.97

Abbreviation: BMI, body mass index; CI: confidence interval; CVD, cardiovascular disease; DBP, diastolic blood pressure; HDL, high-density lipoprotein; LDL, low-density lipoprotein; MRS, metabolic risk score; OW, overweight; OB, obese; OR: odds ratio; SBP, systolic blood pressure; TG, triglycerides; WC, waist circumference.

Adjusted for gender (in total), age, pubertal stage, SES, parental BMI, and parental smoking.

*: p<0.05.

**: p<0.01.

Table 4 describes the association between residence location and increasing MRS and decreasing CRF tertiles. Children in the rural communities, in comparison to their urban counterparts, were associated with an increased odds for CRF classification in the 2^nd^ (OR:2.65, 95%CI:1.7–4.14) and 3^rd^ (OR:2.17, 95%CI:1.34–3.51) tertiles. Children in rural communities were associated with a progressive elevation in odds for MRS classification in the 2^nd^ (OR:1.74, 95%CI:1.11–2.72) and 3^rd^ tertiles (OR:2.42, 95%CI:1.54–3.81).

**Table 4:** Associations between location and decreasing CRF tertiles and increasing MRS tertiles.

	Q1	Q1	Q1
	OR(95%CI)	OR(95%CI)	OR(95%CI)
CRF	1.00 (Reference)	2.65 (1.70–4.14)**	2.17 (1.34–3.51)**
MRS	1.00 (Reference)	1.74 (1.11–2.72)*	2.42 (1.54–3.81)**

Abbreviation: CI, confidence interval; CRF, cardiorespiratory fitness; MRS, metabolic risk score; OR, odds ratio.

Adjusted for gender, age, pubertal stage, SES, parental BMI, and parental smoking.

*: p<0.05.

**: p<0.01.

## Discussion

With China’s rapid economic growth, changes in healthcare delivery, dietary intake, and physical activity patterns have occurred [6]. The results of such alterations are demonstrated by an increasing prevalence of obesity and MetS in low socioeconomic populations [17, 27]. The purpose of this study was to assess the prevalence and associations of childhood CVD risk factors in both Chinese urban and rural communities. Our study found that, compared with urban children, rural children had a higher prevalence of CVD risk factors, specifically hypertriglyceridemia, elevated MRS, and decreased CRF. Furthermore, rural boys had elevated FBG, TG, MRS, and decreased CRF, while urban boys had elevated DBP and LDL level. Rural girls had elevated BMI, FBG, TG, and MRS, as well as decreased CRF. Weight status showed an opposing gender-specific association between rural and urban dwellers. Rural fathers were significantly less likely to have OW/OB weight status as compared to urban fathers, while rural mother were at greater odds of having OW/OB weight status, in contrast to their urban counterparts. Amongst the offspring, similar oppositional gender-specific odds for elevated weight status were found.

The increased prevalence of dyslipidemia amongst rural children found in this study may be attributed to a variety of behavioral, environmental, and dietary factors. Risk factors for childhood dyslipidemia include excessive intake of fats and sugars, genetic predisposition, and low physical activity, etc [28]. Although energy intake was not collected, salt consumption in rural areas has been found to be higher than urban communities, with average salt intake in some rural areas as high as 14.7g, over twice the recommended intake according to Chinese dietary guidelines [29]. Clinical trials and community interventions to reduce salt consumption have also been conducted in rural areas to prevent high blood pressure amongst children and adults [30, 31].

Our study found that the prevalence of hypertensive SBP was higher amongst children of rural areas, in comparison with urban children, which was consistent with Chen *et al*. [8]. In their study, rural children (6–9 years old) and adolescents (10–18 years old), were at greater odds for having elevated BP, in comparison to urban children and adolescents [8]. The China National Nutrition and Health Survey, conducted in 2002, found that rates of familial aggregation of hypertensive SBP was higher amongst rural districts (7.1%), as compared to small/medium-sized cities (6.5%) and major cities (6.3%) [32].

In the analysis of CRF tertile, children of rural areas had greater odds of being in the 2^nd^ and 3^rd^ tertile, as compared to their urban counterparts, indicating poorer oxygen supply to skeletal muscles during sustained physical activity. To our knowledge, this is the first study to compare CRF levels amongst rural and urban Chinese children. When compared to international studies, levels of CRF amongst children in different locations are conflicting. In studies conducted in Spain and Switzerland, CRF was higher in children living in a rural environment, as compared to the urban environment [33, 34]. In national-level studies within the United States and New Zealand, the frequency of physical activity, which is positively associated with CRF, did not differ between rural and urban children [35, 36]. Different CRF amongst urban-rural children of international communities may be attributable to the complex interplay of cultural norms, the built environment, economic development, and government policy.

MetS indicates the aggregation of cardiovascular disease risk factors and has been associated with increased risk of CVD [37]. In the present study, children of rural areas were associated with increased risks of elevated MRS, in comparison to urban children. With such findings, we are unable to validate and compare to other studies within China due to lack of published research in this area. However, in the Swiss study discussed previously, conflicting results were found, as rural Swiss children had significantly decreased MRS, in comparison to urban children [33].

The opposing gender-specific associations of urban-rural weight status, presented in this study, are a well-studied phenomenon in Chinese adults [6]. Fathers in rural locations were less likely to have OW/OB weight status, as compared to urban fathers; whereas rural mothers were at greater odds of having OW/OB weight status, as compared to urban mothers. Similar to our results, studies of Korean urban-rural populations have shown the same gender-specific weight status associations [38, 39]. Our study, unlike any previous study, found the same gender-specific odds of weight status elevation amongst the offspring population. This gender-specific pattern amongst both offspring and parents provides support for the intergenerational relationship between parent and offspring weight status [13, 24].

Several limitations should be considered in the present study. Due to the nature of the cross-sectional study design, causal relations can not be established. Lifestyle factors, including physical activity and urban-rural environmental determinants (such as transportation, healthcare access, and air quality) were not assessed. Misclassification of parental BMI categories might occur because their height and weight were self-reported. Nevertheless, the self-report of these data have been found to have high sensitivity and specificity among young adults [40]. Only one school in each district was included in this study. Therefore, it is likely that the variability of the observations from different schools that occurs would not be captured. Furthermore, due to the study’s exclusive geographic locations, our findings might not be generalizable to children of the provincial or the national level.

In conclusion, this study indicates significant associations between rural residence and several CVD risk factors amongst Chinese schoolchildren. It is, therefore, important to provide interventions aiming at China’s urban-rural healthcare inequity and community-based approaches that reduce familial CVD risk. Nevertheless, the study population is small and likely non-representative. Further large-scale investigations are needed to determine the effects of several important social factors, such as income, lifestyles, nutrition, education, etc. on CVD risks.
